# Risk of Gestational Diabetes Due to Maternal and Partner Smoking

**DOI:** 10.3390/ijerph19020925

**Published:** 2022-01-14

**Authors:** María Morales-Suárez-Varela, Isabel Peraita-Costa, Alfredo Perales-Marín, Agustín Llopis-Morales, Agustín Llopis-González

**Affiliations:** 1 Unit of Public Health and Environmental Care, Department of Preventive Medicine, University of Valencia, 46100 Burjassot, Spain; ipecos@alumni.uv.es (I.P.-C.); allomo@alumni.uv.es (A.L.-M.); agustin.llopis@uv.es (A.L.-G.); 2 CIBER Epidemiology and Public Health (CIBERESP), 28029 Madrid, Spain; 3 Department of Obstetrics, La Fe University Polytechnic Hospital, 46026 Valencia, Spain; Perales_alf@gva.es; 4 Department of Pediatrics, Obstetrics and Gynecology, University of Valencia, 46010 Valencia, Spain

**Keywords:** pregnancy, smoking, passive, prevalence, gestational diabetes, primary care, prevention

## Abstract

Pregnant women are among the most vulnerable to environmental exposure to tobacco smoke (EET); which has been linked to problems in the mothers’ health; one of the most frequent is gestational diabetes (GD). For this reason, there are specific interventions and prevention strategies designed to reduce this exposure risk. However, currently, they are mostly aimed only at aiding the pregnant women with smoking cessation during pregnancy and do not assess or address the risk from passive exposure due to partner smoking. The aim of this work is to study the exposure to EET of pregnant women considering active and passive smoking and to evaluate its effect on the development of GD. This is an observational case-control study within a retrospective cohort of pregnant women. Information on smoking habits was obtained from both personal interviews and recorded medical history. In total, 16.2% of mothers and 28.3% of partners declared having been active smokers during pregnancy; 36.5% of the women presented EET during pregnancy when both active and passive smoking were considered. After adjustments, the association with the EET and GD of the mother was (aOR 1.10 95% CI: 0.64–1.92); for the EET of the partner, it was (aOR 1.66 95% CI: 1.01–2.77); for both partners, it was (aOR 1.82 95% CI: 1.15–2.89), adjusted by the mother’s age and body mass index. There is a lack of education regarding the effects of passive exposure to tobacco smoke. It is essential that pregnant women and their partners are educated on the risks of active and passive smoking; this could improve the effectiveness of other GD prevention strategies.

## 1. Introduction

While the prevalence of smoking has declined in males in most parts of the European Union, in women it seems to have stabilized [1]. Smoking is one of the greatest threats to public health in developed and developing societies [2]. It continues to be the leading cause of preventable death worldwide [2]. Tobacco causes more than 8 million deaths globally every year [3]. Around 15% or 1.2 million of those deaths are non-smokers who have been exposed to second-hand smoke [3]. According to data from the National Health Survey in 2017, 22% of the Spanish population smoked daily [4]. In the EU this proportion was slightly lower at 19% [4]. In Spain, there are more than 52,000 tobacco-related deaths per year [5]. Environmental exposure to tobacco smoke (EET) is the combination of active and passive smoking and is the most common form of exposure to an important environmental pollutant. 

EET is also the most important modifiable risk factor for adverse health outcomes in newborns and children. Pregnant women are among the most vulnerable to exposure to atmospheric environmental pollution, which has been linked to alterations in fetal development [6,7]. Previous studies have reported that tobacco smoking during pregnancy is associated with increased risks of a number of short and long term health complications for both mother and child [8,9,10,11,12,13,14,15,16,17]. Passive EET is associated with numerous harmful effects [2] however; passive EET during pregnancy has not been extensively studied [11,18,19]. Previous studies have demonstrated that pregnant smokers usually have partners who smoke during pregnancy [12,20]. The health of pregnant women and their fetuses is inherently threatened by both active smoking by the mother and passive smoking of the pregnant women’s partners or families [8,20]. 

This highlights the need for prevention strategies, including interventions of a political nature, to reduce the tobacco use. In Spain, the first “anti-smoking law”, with the aim of reducing tobacco use, was passed in 2006 [21] and in 2010, it was modified [22] to include a ban on smoking in any public space that was not outdoors.

Smoking cessation has a positive impact on the trajectory of these health problems, especially if achieved within the first 20 weeks of gestation [23]. Consequently, it is necessary to implement smoking prevention strategies. Intervention programs, including nicotine replacement therapy (NRT), appear to be somewhat effective in helping pregnant women quit smoking. A study determined that, in another hospital of the Valencian Community, 30% of pregnant women who underwent a smoking cessation treatment were able to quit smoking [24]. While most programs to reduce exposure to tobacco during pregnancy are directed exclusively at pregnant women regardless of the environment, their main exposure being living with their partner [25,26,27], there are programs designed to help partners and other household members quit smoking during pregnancy [28]. There is no evidence about the role that exposure to tobacco from the partner of the pregnant woman can play in her exposure. 

Gestational diabetes (GD) is a condition in which carbohydrate/glucose intolerance develops or is first recognized in pregnancy [29]. While the prevalence of GD is extremely variable, it has increased in the last decades by anywhere from 10 to 100%; it is estimated to affect about 6.0 % of pregnant women [30,31,32,33]. The risk of miscarriage and stillbirth increases with worsening glycemic control [34,35,36,37,38,39]. Women with GD are at a higher risk of developing pre-eclampsia, which can lead to pregnancy complications if not treated and polyhydramnios, which can cause premature labor or problems at delivery [40,41]. For the mother, developing GD means being at an increased risk of developing type 2 diabetes in the future [42,43,44].

Previous studies have shown that active smoking is associated with an increased risk of GD in women [45,46]. Passive smoking, on the other hand, has been shown to be associated with an increased risk of type 2 diabetes mellitus, which is caused by insulin resistance and progressive loss of β-cell function and mass [47,48,49,50]. Both insulin resistance and impaired β-cell function contribute to the development of GD [51]. While some studies point to an association between passive smoking and GD [51], the effects of passive smoking on GD are not currently fully established. However, given that GD and type 2 diabetes mellitus share common risk factors and pathogenesis, it is highly plausible that passive smoking may also increase the risk of GD. Previous studies have shown the dose relationship between tobacco consumption and diabetes risk, mainly due to reduced β-cell function [52,53]. Furthermore, inflammatory processes and oxidative stress may increase insulin resistance [54,55]; this may interact with passive smoking to impair β-cell function to amplify the risk of GD.

The children of women with GD are at increased risk of macrosomia, neonatal hypoglycemia, fetal hyperinsulinemia, hyperbilirubinemia, shoulder dystocia, and birth trauma [30,56,57]. The latter two are associated with the baby growing larger than usual, which in turn may lead to difficulties during the delivery and increases the likelihood of needing induced labor or a caesarean section as birth is recommended to occur by the 39th completed gestational week [30,58,59]. The risk of abnormalities of heart and central nervous system are increased; while sacral agenesis is uncommon, it is pathognomic of diabetes [37,60,61,62]. There is an increased risk of the child developing obesity and/or diabetes in later life due to fetal programming [63,64]. 

Given the potential adverse effects on both mother and child, it is imperative to identify risk factors for the developing of GD, in particular those that may be modifiable. There is evidence that links GD to maternal age, ethnicity, socioeconomic status, body mass index (BMI), and family history of diabetes [65,66,67]. However, studies focusing on the role of smoking as a risk factor for GD are limited and conclusions are inconsistent [68,69].

This work intends to carry out an integrated study of the EET of women during pregnancy. The main objectives are to study the EET of pregnant women, taking into account both active and passive smoking and to evaluate the relationship between EET and GD. The aim of this paper is to assess the exposure to EET during pregnancy exclusively due to the mother, compare it with total EET exposure and determine if the smoking habit of the partner modifies the magnitude of the mother’s exposure and risk of GD. In addition, it is intended to identify a risk profile and smoking pattern of higher exposed women. In order to optimize interventions to help reduce exposure to EET, it is important to know whether the passive exposure of the pregnant woman to the partner’s smoking habit should be taken into account when considering programs for the promotion of the cessation of tobacco use and/or the reduction to EET during pregnancy. 

## 2. Methods

The study is observational retrospective cohort and was carried out through a review of the clinical history of the follow-up during the nine months of pregnancy, carried out by specialists in gynecology. The analyses carried out, in which at least one control was carried out in each trimester, were completed with a survey performed on the mother and partner after childbirth. It was made up of pregnant women who belonged to the catchment area of the La Fe Hospital in Valencia, gave birth between February 2017 and April 2020, and were admitted according to the established protocol (Ethic Committee N° 2014/0116). This study was not limited to live births; it included stillbirths but did not include miscarriages before 25 weeks. Participants provided written consent for participation in this study. 

### Measurement of Variables

Smoking during the pregnancy: They were collected from the medical history, specifically from all the controls carried out during the pregnancy, which are at least one medical check-up for each trimester of the pregnancy and completed with information on the delivery and postpartum status of the mother and the newborn. Smoking and other characteristics of the woman and partner were collected at a time shortly after birth; however, the information collected retrospectively assessed their cigarette smoking for the total period of pregnancy. In addition, a face-to-face interview was conducted to complete information of style of life if it did not appear in the pregnancy history.

If the partner was in the hospital, the partner was asked directly; if he was not, the questions corresponding to the partner were answered by the mother. All mothers who gave consent to participate were included during the study period. As an inclusion criterion, it was required that all mothers included in the study signed an informed consent, approved by the Clinical Research Ethical Committee of the Hospital La Fe, which included a confidentiality agreement of the data collected according to Organic Law 15/1999, of 13 December, of Protection of Data of Official Nature.

Those mothers/partners who did not consent or in whom the responses were inconsistent or incomplete were excluded. 

**Figure 1:**
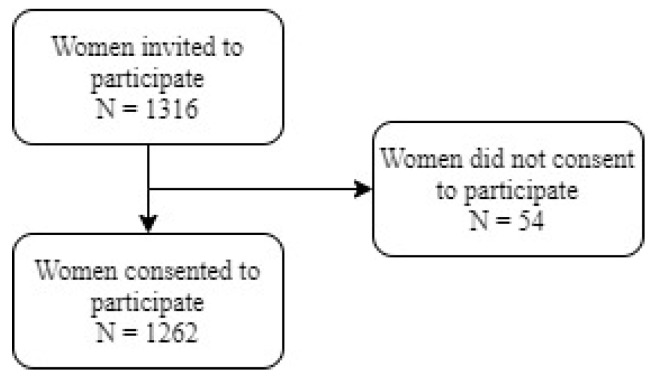
Recruitment of participants.

Participation was offered to a total of 1316 women, 1262 women signed informed consent and were included in the study, giving a participation rate of 95.9%. (Figure 1).

Data was collected using a questionnaire administered during a direct and personal interview face-to-face with the women and partner. The collected information was verified, in order to ensure its quality, validity and completeness and digitized, coded and entered into the IBM SPSS Statistics for Windows, Version 26.0. Armonk, NY, USA: IBM Corp database.

The information collected included personal data, such as the woman’s name, age at the time of delivery, origin or nationality, clinical history number, work situation, level of physical activity at work or at home, as well as at leisure, level of education of both the woman and partner, and, finally, if the woman constituted a family unit together with the other parent of the newborn. Information on parity, number of children and number of previous miscarriages was collected.

The O ’Sullivan test is a test designed to assess blood sugar levels to diagnose cases of GD. In Spain, all pregnant women are routinely given this test between weeks 24 and 28 of pregnancy. In the Valencian Community, the test is also performed between weeks 10 and 13 and/or between weeks 32 and 34 if there are risk factors [58,59]. Blood is drawn and blood glucose is measured. Next, the pregnant woman should ingest a liquid that contains 50 g of sugar dissolved in water; an hour later, blood is drawn again to measure again the blood glucose [70,71].

Blood glucose should be less than 140 mg/dL in the two extractions. If the results offer figures equal to or greater than 140 mg/dL, one can suspect an intolerance to carbohydrates or a GD. GD is diagnosed when the results equal or exceed 200 mg/dL; in this case, it is necessary to repeat the test to confirm it. If the levels obtained have not reached 200 mg/dL, but have matched or exceeded 140 mg/dL, to confirm them the glucose curve or oral glucose tolerance test is performed. In this test, the glycemia values are monitored after an oral overload of 100 g of glucose and four measurements are made at one-hour intervals. The plasma glucose values for each interval should be within these maximum limits: 0 min: 105 mg/dL; 60 min: 190 mg/dL; 120 min: 165 mg/dL and 180 min: 145 mg/dL. If there is a value that exceeds the limits, the test is repeated in three weeks. If the limit is exceeded, glucose intolerance is diagnosed. If two values that exceed the limits appear, GD is diagnosed [70,71].

Information about tobacco use of both the woman and partner in the presence of the mother was collected through a series of questions. Information on the consistency of smoking throughout the pregnancy, number of cigarettes smoked daily and use by the woman of nicotine substitutes was collected. Smoking women were asked if they had received professional advice to stop smoking.

Information on daily number of cigarettes smoked in the three trimesters of pregnancy, both by the woman and the partner in the presence of the mother, was integrated and transformed into a numerical value using the following equations:

First, the EET due to the active consumption of cigarettes by the mother “EETM” was taken into account.

EETM = [ (n° cigarettes/day mother) 1st trimester + (n° cigarettes/day mother) 2nd trimester + (n° cigarettes/day mother) 3rd trimester]/3(1)

Equation (1). Average EET due to active smoking throughout pregnancy.

EET due to the active consumption of cigarettes by the partner in the presence of the mother “EETP” was subsequently considered.

EETP = [ (n° cigarettes/day partner) 1st trimester + (n° cigarettes/day partner) 2nd trimester + (n° cigarettes/day partner) 3rd trimester]/3(2)

Equation (2). Average EET due to passive smoking throughout pregnancy.

Total EET “EETT” was calculated taking into account the number of cigarettes smoked by both woman and partner in the presence of the mother during the gestation period, considering the daily amount smoked in each of the trimesters. This calculation was weighted, considering that the influence of the tobacco smoked directly by the woman is greater than that of the tobacco smoked by the partner in the presence of the mother, so that the woman’s cigarettes were given a value of “1”, while the partner’s were given a value of 0.35. This value was calculated using the information collected regarding the amount of time that the woman and the partner spent in each other’s presence and not sleeping during the week and at the weekend. For this sample population, the average time spent awake together was 35.25% of the day during the total pregnancy.

This way, to carry out the analysis proximity, intensity and duration of exposure are taken into account, integrating all this information into an approximate indicator of actual exposure to tobacco smoke during the gestation period of mother and partner and together. The resulting equations used are shown below:

EETT for each trimester = 1 ∗ EETM + 0.35 EETP = 1 ∗ (n° cigarettes/day mother) × trimester + 0.35 ∗ (n° cigarettes/day partner) × trimester(3)

Equation (3). Average EET due to active and passive smoking for a given trimester.

EETT for pregnancy = 1 ∗ EETM + 0.35 EETP = 1 ∗ [((n° cigarettes/day mother) 1st trimester + (n° cigarettes/day mother) 2nd trimester + (n° cigarettes/day mother) 3rd trimester) /3] + 0.35 ∗ [((n° cigarettes/day partner) 1st trimester + (n° cigarettes/day partner) 2nd trimester + (n° cigarettes/day partner) 3rd trimester)/3](4)

Equation (4). Average EET due to active and passive smoking throughout pregnancy.

EETM and EETT were then compared to assess the importance of passive exposure to tobacco smoke on the level of EET of the pregnant women.

The results obtained from the equation were broken down into percentiles of EETT in order to elaborate the three groups of environmental exposure (“Low”, “Moderate”, and “High”). The “Low” group includes the pregnancy and the partner in the ˂25% of EET (<10 cig./day), the “Moderate” group encompassed from the 25% to the 75% of EET (10–31.5 cig./day) and finally the “High” group represents the women in the >75% of EET (>31.5 cig./day). 

EET was used to study any possible relation between environmental exposure and the sociodemographic characteristics and cigarette consumption habits of the sample. It was also used to evaluate the relationship between environmental exposure and the risk of developing GD during pregnancy. In order to determine the association between EET during pregnancy and GD, binary logistic regression models were applied, by calculating the odds ratio (OR), crude (cOR) and adjusted (aOR) by the women’s age and BMI. The confidence interval (CI) applied was 95%, with a significance of *p* < 0.05. In the regression analyses, the categorical measure of EET was used. The attributable risk and attributable fraction among the exposed were calculated.

## 3. Results

Even though the women and partner were made aware of the risk that smoking during pregnancy carries, 16.2% of women still declared having been active smokers during their pregnancies while 28.3% of the partners declared to be active smokers during the pregnancy and also declared to have done so in the presence of the woman. The combination of both maternal and partner smoking resulted in a total of 36.5% of the women being exposed to tobacco smoke during pregnancy. (Table 1)

The result of integrating the daily number of cigarettes consumed in the three trimesters of pregnancy, both by the woman and the partner, and their categorization into exposure levels, are shown in Table 2. When only taking into account the EET due to active smoking by the mothers, 97.0% (1224/1262) of the women fall into the “No” or “Low” EET groups, with none in the “High EET”. For the EET of the partners, which would represent the passive exposure that the mothers suffer, the number of individuals in the “No” or “Low” EET groups fell to 83.3% (1051/1262). After integrating both the active and passive exposures, the number of women in the “No EET” group was 63.5% (801/1262); in the “Low EET” group, it was 9.2% (116/1262); in the “Moderate EET” group, it was 18.3% (231/1262); finally, in the “High EET” group, it was 9.0% (114/1262).

Of the women studied, 205 (16.2%) smoked actively at some point during pregnancy. The most common daily cigarette consumption was 1–5 cigarettes. Only 4 (0.3%) women consumed nicotinic substituents. Most notable is the fact that only 2.1% of smoking women reported having been professionally counseled by medical staff to stop smoking during pregnancy.

Of the women who smoked, 43.4% (89/205) had a “High EET”, 41.4% (85/205) had a “Moderate EET” and 15.1% had a “Low EET” (31/205). Of the women who reported not having smoked during pregnancy, 8.0% (85/1057) had “Low EET”, 13.8% (146/1057) had a “Moderate EET and 2.4% (25/1057) had a “High EET”. There is a coincidence between “High EET” and a higher intensity of smoking, with women who consumed 5 to 10 cigarettes accounting for the highest percentage (~50%) of the “High EET” group. On the other hand, women who consumed nicotinic substituents during gestation are split evenly into the “Low” and “High” EET levels.

28.3% of partners smoked actively at some point during pregnancy. There is a slight decrease in the daily number of cigarettes consumed by the partners as the pregnancy progresses. Women with a partner who consumes 10–20 cigarettes per day were more likely to fall into the “High EET” group accounting for about 58% of the women in the group. Meanwhile, having a partner who consumes 5 to 10 cigarettes per day was a predictor for “Moderate EET” as about 50% of women in the group met this criterion.

The characteristics of the woman according to the different levels of EET are shown in Table 3. Most women were between the ages of 31 and 40 (58.6%). The average age of the participating women is just over 32 years old.

Women over 41 years of age had the highest percentage of “No EET”, (50/67, 74.6%), while the most exposed would be those under 20 (20/39, 51.3% in “No EET”). For “High EET” level, the highest percentage appeared in women between 21 and 30 years old (58/114, 50.9%). There were significant differences for EET according to age.

The majority of participating women were of western European origin (1048/1262, 83.0%), most of which were of Spanish nationality. Women from Eastern Europe were the most environmentally exposed (54.5%, 24/44), with 11.4% (5/44) in the “Low EET”, 22.7% (10/44) in the “Moderate EET”, and 11.4% (5/44) in the “High EET”, followed closely by women from Western Europe. 

Only 1.5% (19/1262) of the women were single mothers or did not constitute a family unit together with the baby’s other parent. In the case of single mothers, significant differences could also be observed, although the number of cases was very small. 

Secondary studies are the most prevalent type of study for both mothers and partners, with 51.0% (644/1262) and 49.2% (621/1262), respectively. The highest percentages of “No EET” occur in the case of women and partners with higher education. The highest percentages of “High EET” are given in women and partners with primary or no studies. There were significant differences for EET according to level of studies.

The majority of mothers reported having spent most of their working hours during pregnancy, sitting or standing without large movements, with percentages of 45.3% and 41.7% respectively. In their free time, most of the mothers reported maintaining a light physical activity (walking, light bicycle), with a percentage of 53.7%, followed by those who did not do any type of physical activity with 27.6%. Only 1.0% of mothers performed tasks that required great physical effort during their workday, and only 0.6% of mothers engaged in intense physical activity in their free time during pregnancy. It is noteworthy that, considering the level of exposure, women performing tasks during the workday that require great physical effort were the most highly exposed; meanwhile, the least exposed mothers correspond to the group with the least physical activity at work, the differences being statistically significant. On the other hand, regarding the level of physical activity in free time, no significant differences were found between the different levels and exposure to the pollutant.

The majority of the participants were primiparae (777/1262, 63.4%). In total, 62.1% (761/1262) of women had only one child, while only 8.1% (99/1262) were mothers of three or more children. No significant differences with respect to parity were observed. There were notable differences with respect to the number of children, with those women in the “Moderate EET” and “High EET” being more likely to have a higher number of children. 

In total, 69.3% (875/1262) reported never having had a miscarriage, and only 2.0% (26/1262) of the participants had previously had more than two miscarriages. There are also statistically significant differences for the number of miscarriages, where the highest number of miscarriages (>2) occurred in women with “High EET”. 

A total of 17.4% (220/1262) of the mothers reported having a preexisting condition of those contemplated in the study. The results did not show statistically significant differences.

Table 4 shows the risk assessment expressed as ORs of GD in relation to the EET and active smoking status of the mother, partner, and both during pregnancy. In the crude assessment, there was an association between developing GD and the EET of the partner (cOR:1.78 95% CI: 1.08–2.93) and total EET (cOR 1.64 95% CI: 1.03–2.61). After adjustment, the association for both the EET of the partner (aOR 1.67 95% CI: 1.01–2.76) and total EET remained statistically significant (aOR 1.82 95% CI: 1.15–2.89). The results for the association between developing GD and smoking status were very similar to those relating to EET.

In the adjusted model and using the maternal EET as the unexposed group, the attributable risk and attributable fraction among the exposed for the passive contribution to exposure were 40% and 26.5%, respectively. This means that the difference between the risk of developing GD when comparing the maternal-only EET to the total EET was 40%. Therefore, 26.5% of the cases of GD were attributable to the passive exposure.

## 4. Discussion

Exposure to tobacco smoke during pregnancy is an important preventable cause of maternal, fetal, and infant morbidity and fetal mortality [60]. Given this, EET is a major public health concern [72], especially in Spain, as it is still above the EU average in the number of smokers [3]. While pregnant women are aware of the negative consequences of smoking during pregnancy, this seems to not be enough to motivate all of them to quit [73,74]. Studies have identified a range of barriers that pregnant women face when trying to quit smoking; relationships can play a significant role [75,76]. A partner’s smoking status can affect the level of exposure of the woman and their attitude can influence a pregnant woman’s attempt to quit [77]. Therefore, interventions should not be limited exclusively to the mother; they must widen their focus to cover the greatest possible number of people in the woman’s environment and should aim to raise awareness in society as a whole. 

In addition to the preventive strategies of each country, at the global level, lines of action have also been established. In 2008, based on the WHO Framework Convention on Tobacco Control, the WHO proposed the MPOWER strategy [78], based on six essential pillars: monitoring tobacco consumption and preventive policies, protecting passive smokers from second-hand smoke—which relates to our study—offering cessation programmers, warning of the dangers of tobacco consumption, reinforcing the probation of advertising campaigns on tobacco consumption and raising the tax burden on tobacco sales. In Spain, the latest policy review of lines of action in accordance with the MPOWER strategy was a 2019 agreement of the Public Health Commission of the Inter Territorial Council of the National Health System.

According to 2009 data, the prevalence of tobacco consumption in women of childbearing age was 27.68% in Spain; however, this is not applicable to pregnant women [79]. The percentage of women who smoke actively during gestation, in this study, 16.2%, coincides with other Spanish studies [80,81] and is higher than those of international studies [82]. Traditionally, in Spain, the prevalence of smoking during gestation has always been higher than in other European countries [83]. The percentage of pregnant women who smoke during pregnancy was lower than in previous Spanish studies [7], which could be due to the result of prevention and awareness policies. 

While the percentage of women who are active smokers during gestation (16.2%) was lower than the partners (28.3%), the prevalence of smoking during a period as delicate as gestation is still considerably high in women. The strong influence of the partner’s smoking habit on the exposure suffered by the woman is significant. Although only 16.2% of women are active smokers during pregnancy, a total of 36.5% are exposed to tobacco. This indicates that 20.3% of the participating women were non-smokers, but, due to their partner’s smoking habit, suffered EET. This effect can also be seen in the women who did smoke during pregnancy. While the majority of the women who smoked during pregnancy fell within the “Low” EET category, and none in the “High” EET category, when partner smoking was taken into account, most smoking women now fell into the “Moderate” EET group, with a few in the “High” EET group. The effect of the partner’s’ smoking habit could also be seen in the results from the adjusted odds ratio calculations. In this case, when the contribution to EET exposure from passive smoking was included, the risk of developing GD was slightly more elevated and, while still relatively low, statistically significant. This leads us to think that there is a lack of health education regarding the effects of passive exposure to tobacco smoke. It is essential that pregnant women and their partners are educated on the health risks of both active and passive smoking and how these could affect the health of their child and their own health [8,12,84,85,86,87].

The profile of pregnant women exposed to tobacco smoke in the present study was that of a woman younger than 30 years old, Spanish, with a smoking partner, low level of education, a job that requires physical effort, multiparous and who has suffered previous miscarriages. 

Smoking during pregnancy not only impacts the woman’s health, but also that of her child. Partners should also be made aware of the associated risk and encouraged to participate in smoking cessation programs. Women who have managed to quit smoking should be helped to remain smoke free after birth, since they are at a high risk of relapsing [88]. 

The study shows that there is a risk of GD developing in women who are exposed to tobacco smoke, compared to those who are not. The existence of the risk of developing GD in women exposed to environmental tobacco smoke coincides with some previous studies, which state that tobacco use during pregnancy is related to the development of GD [45,89,90,91,92,93]. However, studies of smoking and GD have yielded inconsistent findings over the years. Possible explanations are that residual confounding factors have caused the erroneous identification of an association between smoking and GD, or that confounding factors and low power have led to the erroneous conclusion that no association exists [45]. 

Two systematic reviews/meta-analyses, one published in 2008 and the other published in 2018, were found on the association between cigarette smoking and GD [68,69]. The 2008 study included 12 studies; only four described aORs and assessed potential confounders when non-smokers were compared to smokers [69]. The combined overall aOR was (0.95; 99% CI:0.85–1.07). A sensitivity analysis excluding a population with a high prevalence of GD found only minimal changes in the aOR (0.97; 95% CI: 0.69–1.38) [69]. The sub-group analysis performed by type of diagnostic criteria did not reveal important differences [69]; however, the sub-group analysis by gestational age of smoking assessment showed that, when smoking was assessed before 24 weeks of pregnancy, the cOR, was (1.22; 99% CI 0.73–2.04), whereas, when it was assessed after 24 weeks, it was (0.88; 99% CI 0.70–1.10) [69].

The 2018 study on the association between GD and cigarette smoking during pregnancy found that the pooled OR was (0.98; 95% CI: 0.88–1.10) for GD, with (1.10; 95% CI: 0.97–1.25) for light smoking and (1.02; 95% CI: 0.67–1.53) for heavy smoking [68]. However, it must be noted that the 12 studies included in that meta-analysis assessed only active smoking by the mother and most only via a dichotomous question dividing women into smokers and nonsmokers. 

Given the results of the present study, where passive smoking has a considerable effect on exposure and risk of GD, it would seem logical that the pooled association found may be underestimated. While it is likely that the present study also underestimates the association due to an underestimation of the passive exposure suffered by the mothers, it presents a more realistic scenario regarding EET. With the results of this study, it could be said that at least 26.5% of the cases of GD are attributable to passive exposure to tobacco smoke. In the future, with studies that more accurately and completely reflect passive exposure, it would be expected that this number could be higher, highlighting the importance of passive smoking in the development of GD. Moreover, given what is known about the effects of tobacco on other diseases, such as respiratory problems or cardiovascular diseases, the possible “multiple effect” of passive exposure to EET on the risk of other pathologies should be considered. 

## 5. Strengths and Weaknesses

There are many differences in the literature regarding smoking habit during pregnancy. Most studies only consider whether or not the woman has smoked during pregnancy [94,95] and take into account the number of cigarettes consumed daily [96,97]. One study differentiates the smoking habit between the three trimesters of pregnancy, although not counting the number of cigarettes consumed [98]. While other studies structured their analysis according to whether the women were not smokers, were ex-smokers, or otherwise smoked during pregnancy [10,99]. One study was found where maternal smoking and secondhand smoke exposure were measured using a series of questions and were then combined into a six-level smoking exposure summary variable [45]. However, classification into each of the levels was based on smoking status (non-smoker/abstainer/smoker) in the 2 years prior to the study and the last 3 months of pregnancy [100]. In summary, these studies mainly only considered exposure due to active smoking; if passive exposure was considered, it was done dichotomously. 

Other authors also studied the passive exposure due to tobacco consumption of other people in the presence of the woman [6,98]. Nonetheless, these studies collected information in a partial way regarding smoking and exposure and are scarce. Therefore, it has been considered desirable to design a new methodology with a new form of categorization of what, in this study, has been called EET.

This is precisely one of the most relevant introductions of this work regarding the revised literature: the consideration of tobacco smoke as an environmental pollutant and the study of its exposure as such. A tool which took into account both the woman’s and the partner’s tobacco consumption in the presence of the woman, as well as the daily number of cigarettes consumed by both in each of the trimesters of gestation, was developed. One of the main innovations of this methodology was the calculation of the total intensity of exposure from the daily number of cigarettes consumed by the parents, which served as an indicator of exposure. Thus, this study was the first to define the exposure in three levels. 

One of the main weaknesses of this study was present within the calculation of the partners’ contribution to environmental exposure. Obtaining reliable information on the precise number of cigarettes that the partners’ smoked in the presence of the mother was difficult. While mothers and partners were able to give information on the total daily number of cigarettes consumed by the partners, when asked how many of those cigarettes were in the presence of the mother, the answers provided were dubious. While, ideally, each couple would have its own value for partner contribution to EET, the difficulty in establishing this value lead to the use of 0.35, as to not over or underestimate the partners’ contribution, for all the cases and the development of one single equation for the evaluation of EET. This could be a source of non-differential (random) misclassification of individual EET exposure and could result in a reduction in the power of the study, thus making it more difficult to detect an association between EET and risk of GD. However, in this case, given that an association was detected, it could mean that it was being undervalued. Misclassification was also likely to be different between mothers and partners, between those diagnosed and those not, and perhaps even by trimester. While most of the bias was most likely toward the null, this is not known for certain and should be considered. 

Despite the great coverage of delivery attendance at La Fe Hospital and its representativeness, the selection of the study population as the women giving birth only in that hospital could be associated to a selection bias by social class, a factor associated with smoking which could lead to an overestimation of the prevalence of smoking. However, given the similarity of the prevalence found here with that of other studies, this limitation could be considered minimal.

The study of EET based solely on self-reported tobacco use can be considered as an inaccurate index of exposure, since it can hide relevant information. Biological determination of substances such as nicotine or cotinine in different fluids is a good option to give robustness to these studies [101,102,103]. The use of biomarkers would require a significant economic investment that is not always feasible and that carries other problems. Although all of the above are limitations, the results obtained on the prevalence of consumption were similar to other studies in which more precise biomarkers have been used, [98,104]; therefore, the results obtained for this work can be considered valid.

The collection of information was subject to the availability of the clinical reports of the woman and the newborn (not always available at the time of the consultation), and if these were correctly filled or, conversely, if information was lacking, which meant that this phase of the study was prolonged in time.

## 6. Conclusions

The results obtained allowed us to identify that the prevalence of smoking during pregnancy was higher in partners than in women and it significantly influenced the woman’s EET during pregnancy. There seems to be a lack of health education regarding the effects of passive exposure to tobacco smoke during pregnancy. There was a slightly increased risk of GD not only in women who had smoked during pregnancy, but also in women who had not actively consumed tobacco but who had been exposed during this period due to the smoking habit of their partner. This information, that partner smoking should be taken into consideration, can be very valuable given the need to adequately target smoking prevention strategies during the pregnancy as a key aspect of their effectiveness in the prevention of GD.

Gestation is a delicate stage, in which any problem or condition can trigger adverse effects, not only on the fetus, but also in the long term, in childhood or even in adulthood. With this in mind, it seems essential to minimize any risk that a woman may suffer during this period, and that may affect both her and the newborn. Not only should the mother be informed about the benefits of smoking cessation, her partner and wider environment should be as well, given its influence on the total exposure of the woman. Interventions cannot be limited exclusively to the mother; they must widen their focus to cover the greatest possible number of people in the woman’s environment and should aim to raise awareness in society as a whole.

## Figures and Tables

**Table 1:** Environmental exposure to tobacco smoke.

	n (n = 1262)	%
**Mother active smoker**		
No	1057	83.8
Yes	205	16.2
**Partner active smoker**		
No	905	71.7
Yes	357	28.3
Mother exposed to tobacco *		
No	801	63.5
Yes	461	36.5

* Mother and/or partner is an active smoker.

**Table 2:** Calculated levels of environmental exposure to tobacco during pregnancy.

	EET Mother		EET Partner		EET Total	
	n	%	n	%	n	%
**No EET**	1057	83.8	905	71.7	801	63.5
**Low EET** (<10 cig./day)	167	13.2	146	11.6	116	9.2
**Moderate EET** (10–31.5 cig./day)	38	3.0	208	16.5	231	18.3
**High EET** (>31.5 cig./day)	0	0	3	0.2	114	9.0

EET: environmental exposure to tobacco smoke.

**Table 3:** Sociodemographic characteristics of the women included in the study.

	Total		Environmental Exposure to Tobacco Smoke During Pregnancy (n = 1262)								
			No EET		Low EET		Moderate EET		High EET		
			(n = 801) (63.5%)		(n = 116) (9.2%)		(n = 231) (18.3%)		(n = 114) (9.0%)		
	n	% ^a^	n	% ^b^	n	% ^b^	n	% ^b^	n	% ^b^	*p*
**Age (years)**											0.001 *
≤20	39	3.1	20	2.5	5	4.3	10	4.3	4	3.5	
21–30	416	33.0	230	28.7	43	37.1	96	41.6	47	41.2	
31–40	740	58.6	501	62.5	63	54.3	118	51.1	58	50.9	
41–50	67	5.3	50	6.2	5	4.3	7	3.0	5	4.4	
**Nationality**											0.001 *
Western Europe	1048	83.0	637	79.5	99	85.3	206	89.2	106	93.0	
Eastern Europe	44	3.5	24	3.0	5	4.3	10	4.3	5	4.4	
North America	3	0.2	3	0.4	0	0.0	0	0.0	0	0.0	
Central America	20	1.6	14	1.7	3	2.6	2	0.9	1	0.9	
South America	96	7.6	78	9.7	6	5.2	10	4.3	2	1.8	
Northern Africa	29	2.3	24	3.0	2	1.7	3	1.3	0	0.0	
West Africa	7	0.6	6	0.7	1	0.9	0	0.0	0	0.0	
Central África	6	0.5	6	0.7	0	0.0	0	0.0	0	0.0	
Eastern Asia	6	0.5	6	0.7	0	0.0	0	0.0	0	0.0	
Western Asia	1	0.1	1	0.1	0	0.0	0	0.0	0	0.0	
South Asia	2	0.2	2	0.2	0	0.0	0	0.0	0	0.0	
**Single mother**											0.041 *
No	1243	98.5	789	97.8	116	100.0	230	99.6	114	100.0	
Yes	19	1.5	18	2.2	0	0.0	1	0.4	0	0.0	
**Maternal education level**											0.001 *
No formal education	33	2.6	18	2.2	2	1.7	8	3.5	5	4.4	
Primary studies	164	13.0	82	10.2	21	18.1	38	16.5	23	20.2	
Secondary studies	644	51.0	375	46.8	65	56.0	135	58.4	69	60.5	
University degree	388	30.7	300	37.5	24	20.7	48	20.8	16	14.0	
Postgraduate degree	33	2.6	26	3.2	4	3.4	2	0.9	1	0.9	
**Paternal education level**											0.001 *
No formal education	63	4.5	41	5.2	4	3.4	7	3.0	5	4.4	
Primary studies	191	15.2	91	11.4	22	19.0	50	21.6	28	24.6	
Secondary studies	621	49.4	367	46.2	63	54.3	128	55.4	63	55.3	
University degree	364	29.0	277	34.8	25	21.6	44	19.0	18	15.8	
Postgraduate degree	23	1.8	19	2.4	2	1.7	2	0.9	0	0.0	
**Maternal employment status**											0.533
Unemployed	184	14.6	112	14.0	14	12.1	39	16.9	19	16.7	
Employed	1078	85.4	689	86.0	102	87.9	192	83.1	95	83.3	
**Physical activity at work/home**											0.001 *
Sitting	572	45.3	383	47.8	49	42.2	93	40.3	47	41.2	
Standing without movement	526	41.7	332	41.4	53	45.7	102	44.2	39	34.2	
Walking, frequent movement, weigh bearing	141	11.2	78	9.7	11	9.5	30	13.0	22	19.3	
Greta physical effort	12	1.0	4	0.5	3	2.6	3	1.3	2	1.8	
Unable to answer	11	0.9	4	0.5	0	0.0	3	1.3	4	3.5	
**Physical activity free time**											0.084
None	348	27.6	205	25.6	31	26.7	71	30.7	41	36.0	
Light	678	53.7	442	55.2	59	50.9	117	50.6	60	52.6	
Moderate	228	18.1	151	18.9	26	22.4	40	17.3	11	9.6	
Intense	8	0.6	3	0.4	0	0.0	3	1.3	2	1.8	
**Parity**											0.674
Primiparae	777	63.4	492	64.2	75	65.2	138	60.0	72	63.2	
Multiparae	448	36.6	274	35.8	40	34.8	92	40.0	42	36.8	
**Total number of children**											0.012 *
1	761	62.1	495	64.6	72	62.6	133	57.8	61	53.5	
2	365	29.8	217	28.3	36	31.3	74	32.2	38	33.3	
>3	99	8.1	54	7.0	7	6.1	23	10.0	15	13.2	
**Previous miscarriages**											0.006 *
No	875	69.3	569	71.0	80	69.0	161	69.7	65	57.0	
Yes	387	30.7	232	29.0	36	31.0	70	30.0	49	43.0	
1	279	22.1	176	22.0	21	18.1	44	19.0	38	33.3	
2	82	6.5	45	5.6	10	8.6	23	10.0	4	3.5	
>2	26	2.0	11	1.4	5	4.3	3	1.3	7	6.2	
**Maternal pathology previous to pregnancy**											0.404
No	1042	82.6	614	82.3	124	85.5	197	80.7	107	84.3	
Yes	220	17.4	132	17.7	21	14.5	47	19.3	20	15.7	

EET: environmental exposure to tobacco smoke, ^a^ Percentage shown calculated for column, ^b^ Percentage shown calculated for row, * *p*-value < 0.05.

**Table 4:** Risk assessment (OR) of effects in pregnancy and adverse events in the newborn in relation to the “EET”.

	Environmental Exposure to Tobacco Smoke During Pregnancy (n = 1262)					
	EET Mother	EET Partner	EET Total	Mother Active Smoker	Partner Active Smoker	Mother Exposed to Tobacco *
Gestational diabetes (n = 106)						
OR	1.20	1.78	1.95	1.22	2.00	1.97
IC 95%	0.70–2.07	1.08–2.93	1.23–3.08	0.71–2.08	1.23–3.25	1.25–3.11
*p*-value	0.488	0.022	0.004	0.471	0.005	0.004
OR ^a^	1.10	1.67	1.82	1.10	1.89	1.84
IC 95%	0.64–1.90	1.01–2.76	1.15–2.89	0.64–1.90	1.16–3.10	1.16–2.93
*p*-value	0.732	0.046	0.011	0.721	0.011	0.010
OR Age ^a^	1.72	1.69	1.66	1.72	1.68	1.66
IC 95%	1.20–2.47	1.17–2.44	1.15–2.38	1.19–2.47	1.17–2.41	1.15–2.38
*p*-value	0.003	0.005	0.006	0.003	0.005	0.006
OR IMC ^a^	1.35	1.36	1.36	1.35	1.39	1.36
IC 95%	1.01–1.80	1.02–1.82	1.02–1.82	1.01–1.80	1.02–1.82	1.02–1.82
*p*-value	0.042	0.037	0.039	0.042	0.039	0.038

EET: environmental exposure to tobacco smoke, * Mother and/or partner is an active smoker, ^a^ Adjusted for characteristics of the mother: age, BMI.

## Data Availability

The data presented in this study are available on request from the corresponding author. The data are not publicly available due to personal data protection issues.
